# The preparation of an ultrastable mesoporous Cr(iii)-MOF *via* reductive labilization†
†Electronic supplementary information (ESI) available. See DOI: 10.1039/c5sc02587g


**DOI:** 10.1039/c5sc02587g

**Published:** 2015-09-02

**Authors:** Xizhen Lian, Dawei Feng, Ying-Pin Chen, Tian-Fu Liu, Xuan Wang, Hong-Cai Zhou

**Affiliations:** a Department of Chemistry , Texas A&M University , College Station , Texas 77842-3012 , USA . Email: zhou@chem.tamu.edu; b Department of Material Science and Engineering , Texas A&M University , College Station , Texas 77843 , USA

## Abstract

Kinetic labilization of the Fe(iii)–O coordination bond in a mesoporous metal–organic framework, PCN-333-Fe(iii), is realized by the reduction of Fe(iii) by Cr(ii). The Fe(ii)–Cr(ii) metathesis is thus completely achieved followed by this reductive labilization process that generates PCN-333-Cr(iii). The kinetic inertness of Cr(iii) provides PCN-333-Cr(iii) with enhanced chemical stability as well as a broader range of applications compared to those of PCN-333-Fe(iii). For instance, alkylamine incorporated PCN-333-Cr(iii) demonstrated significant carbon dioxide uptake while PCN-333-Fe(iii) lost its crystallinity after alkylamine treatment.

## Abstract 2

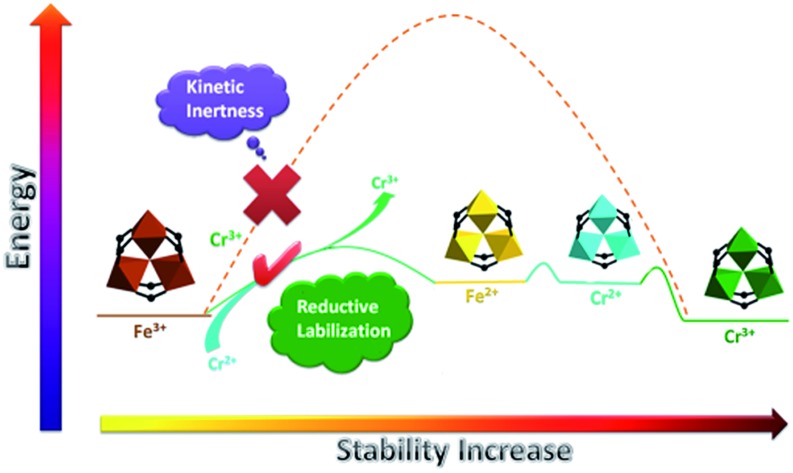
A novel reductive labilization–metal metathesis method is reported for the synthesis of an ultrastable mesoporous Cr(iii)-MOF.

## Introduction

Bearing high surface area, large pore size and volume as well as the tunability of pore environments and functionalities, metal–organic frameworks (MOFs) have demonstrated promising applications in gas storage, separation, catalysis, guest moiety immobilization, drug delivery and sensing.^1^–^5^ Many of these applications include the use of metal nodes, or secondary building units (SBUs), as active sites. SBUs are always explored as Lewis acid species, while their redox properties have been studied in only a few reports.^6^,^7^ One of the experimental demonstrations is the reduction of high valence Fe(iii) to generate low valence Fe(ii) in MIL-100-Fe(iii) by carbon monoxide, indicating the possibility of tuning the chemical robustness of the framework by a redox reaction.^7^

**Fig. 1:**
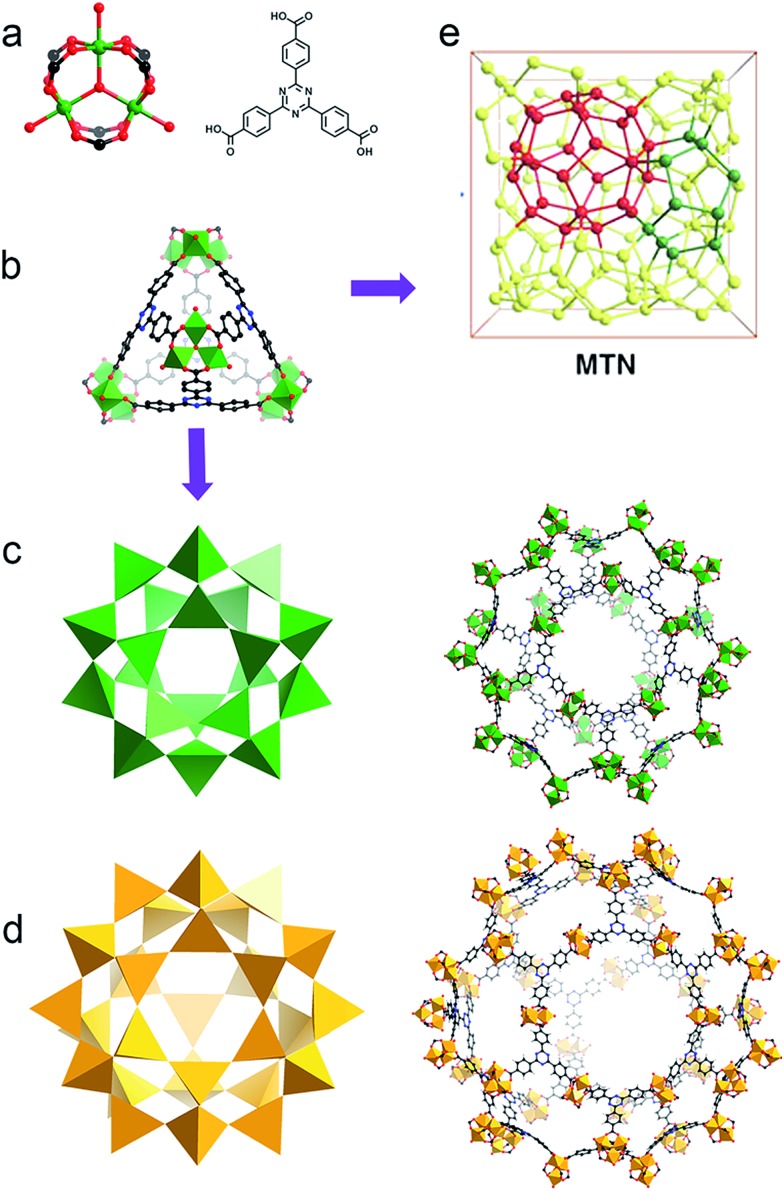
(a) PCN-333 is composed of trimeric clusters and TATB ligands with (b) supertetrahedra as supermolecular building blocks. (c) The small cage with a diameter of 4.2 mm and (d) the large cage with a diameter of 5.5 nm in the construction of (e) the network with MTN topology.^8^

Based on the Hard and Soft Acid and Base (HSAB) principle,^28^ chemically robust MOFs can be constructed with carboxylate ligands, hard Lewis bases, and high valence metal ions, such as Fe(iii), Cr(iii) or Zr(iv), categorized as hard Lewis acids. Compared with MOFs composed of divalent species, many of these high valence metal-containing MOFs can survive in water, or even acid or base solutions. This phenomenon has been extensively demonstrated in the MIL series, UiO series and PCN-22X series.^9^–^15^,^26^ A recent example is an iron based mesoporous MOF, PCN-333-Fe(iii), which is stable in both acidic and basic aqueous solutions despite its ultrahigh porosity (Fig. 1).^8^ However, MOFs constructed with high valence metal ions may suffer from structure breakdown in some specific environments, for instance, PCN-333-Fe(iii) totally loses its crystallinity and porosity in alkylamine solution. Incorporation of kinetically inert metal ions, for example, Cr(iii), into the framework backbone could generate MOFs with exceptional stability.^16^,^26^

Nevertheless, obtaining crystalline Cr(iii)-MOFs with carboxylate ligands are extremely difficult due to the kinetic inertness of Cr(iii). Hydrothermal conditions are exclusively adopted in the synthesis of crystalline Cr(iii)-MOFs, but these conditions might be unfeasible when using larger organic ligands as they are extremely hydrophobic. Attempts to synthesize PCN-333-Cr(iii) using either hydrothermal or solvothermal conditions with temperatures as high as 220 °C failed to generate any crystalline products. An alternative synthetic pathway is the post synthetic metathesis of metal clusters from a template MOF with a known structure. Fe(iii)-MOFs can serve as practical structural templates because iron resembles chromium in both coordination geometry and valence. Disappointingly, the metathesis of PCN-333-Fe(iii) with CrCl_3_ for 24 hours only yielded partially metathesized MOF. The incompleteness of the above metathesis is not surprising since in several reports demonstrating the feasibility of metal metathesis for high valance MOFs, complete metal metathesis has never been achieved.^24^ This is mainly attributed to two reasons: (a) the dissociation of high valence metal ions from a framework is thermodynamically unfavorable; (b) the dissociation rate of high valence ions is much slower than that of divalent species due to the much higher activation energy.^25^ Therefore, a long reaction time or elevated reaction temperature is required in order to achieve complete metathesis. However, under these scenarios, framework decomposition is usually inevitable due to the acidic environments generated by high valence species.^17^,^25^

Herein, we report a reductive labilization–metathesis route for the synthesis of PCN-333-Cr(iii) using PCN-333-Fe(iii) as the template, wherein redox chemistry contributes to the generation of labile metathesis intermediates. PCN-333-Cr(iii) demonstrated a broader range of applications than PCN-333-Fe(iii) in consequence of its improved chemical stability. Alkylamine incorporated PCN-333-Cr(iii) demonstrated significant CO_2_ adsorption capacity at low pressure whereas PCN-333-Fe(iii) barely shows any CO_2_ adsorption capacity due to structural decomposition in alkylamine solution.

## Results and discussion

MOFs constructed with divalent metal ions have been demonstrated to be good templates for complete metal metathesis due to the kinetic lability of their coordination bonds.^17^–^23^,^29^ The reduction of high valence metals in SBUs can labilize the inert MOF, which will provide the possibility for complete metathesis using an inert MOF template. There are several prerequisites for this process to take place: (1) the metal ions of the framework are in their high oxidation state and can be readily reduced under mild conditions; (2) the reductant will not generate harsh conditions after oxidation (for example, very low/high pH values); (3) the redox potential of the oxidant is much higher than the redox potential of the reductant, resulting in an irreversible redox reaction. In the practical case, PCN-333-Fe(iii) is composed of an oxidative Fe(iii) species while CrCl_2_ matches the prerequisites to be a suitable reductant. The M^3+^/M^2+^ redox potentials for Fe and Cr in aqueous solutions are: 0.77 V (Fe), –0.42 V (Cr).^25^ The large potential difference indicates that a redox reaction can irreversibly take place between Fe(iii) and Cr(ii) as indicated in eqn (1):

1 Fe^3+^ + Cr^2+^ → Fe^2+^ + Cr^3+^

Driven by the concentration gradient, the metal metathesis between Fe(ii) in the intermediate framework and Cr(ii) in the solution is favorable.

**Fig. 2:**
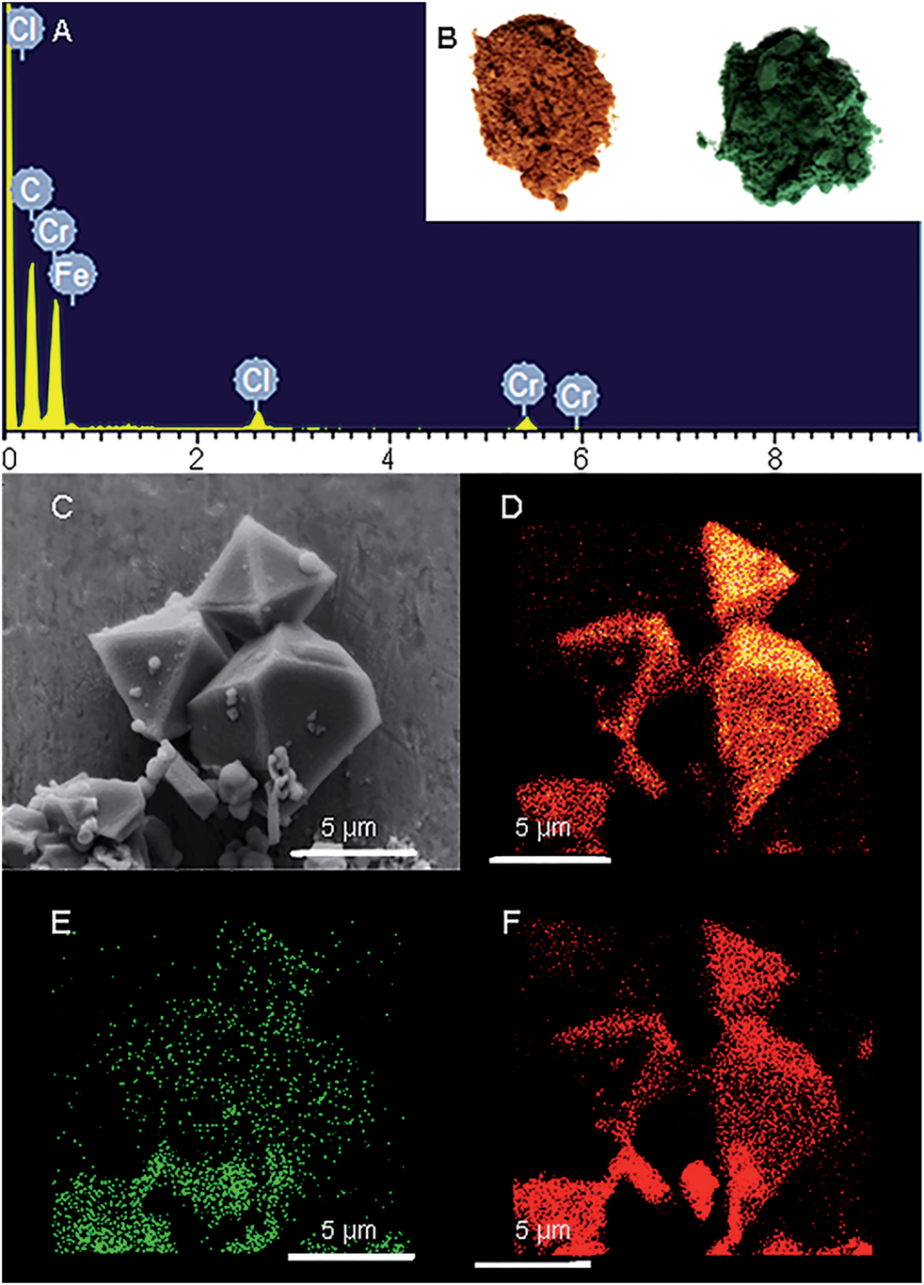
(A) The EDS spectrum of PCN-333-Cr(iii); (B) PCN-333-Fe(iii) on the left and PCN-333-Cr(iii) on the right; (C) SEM images of PCN-333-Cr(iii); elemental mappings of (D) C, (E) Cr, and (F) O from EDS analysis on PCN-333-Cr(iii).

**Fig. 3:**
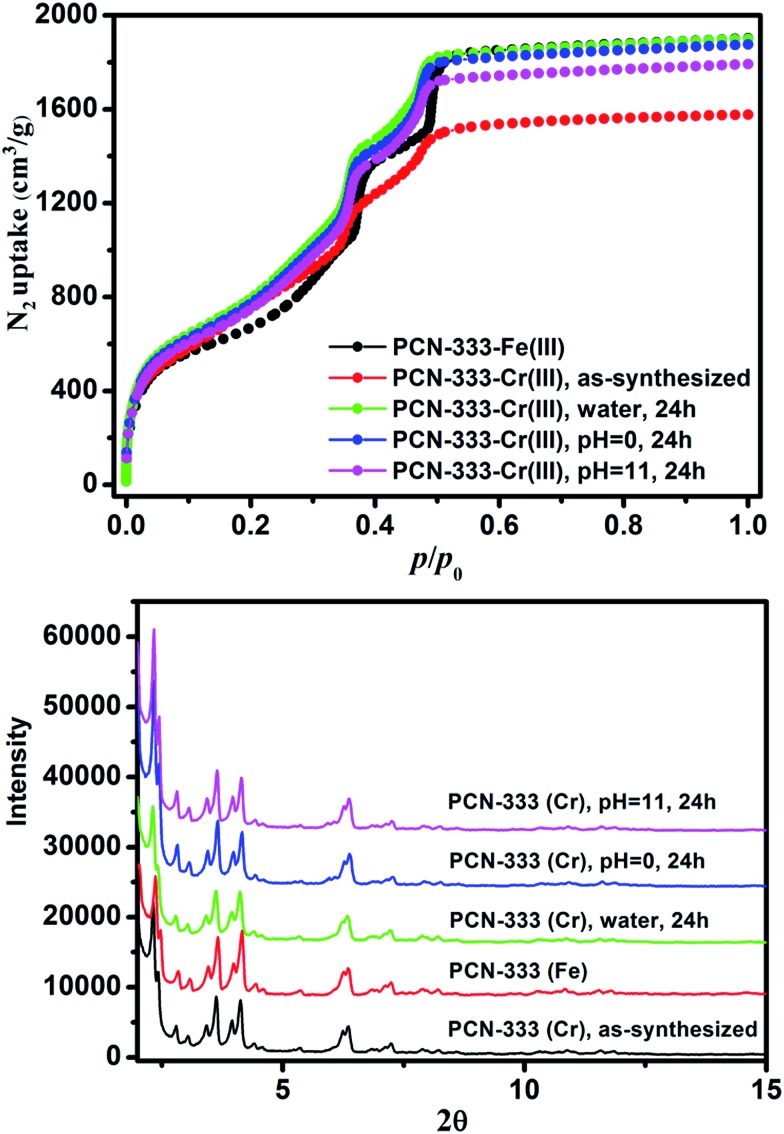
N_2_ isotherms (top) and PXRD patterns (bottom) of PCN-333-Fe(iii), PCN-333-Cr(iii), PCN-333-Cr(iii) treated with water, pH = 0 and pH = 11.0 aqueous solutions for 24 hours.

Freshly prepared PCN-333-Fe(iii) was dispersed in a solution of CrCl_2_ in dry *N*,*N*-dimethylformamide (DMF) at 85 °C under the protection of N_2_. The color of the solid turned gradually from reddish brown to deep green (Fig. 2B). The complete metathesis of Fe by Cr was obtained after 30 minutes as confirmed by inductively coupled plasma mass spectrometry (ICP-MS) and energy-dispersive X-ray spectroscopy (EDS) (Fig. 2A and Table S1†). The SEM-EDS mapping with 4 000 000 counts results indicated that Cr, C, O and Cl were uniformly distributed on the crystal surface (Fig. 2D–F and S2†). After that, the solids were washed with DMF three times in air to guarantee that all of the Cr(ii) ions in the framework were oxidized to Cr(iii), which was verified using X-ray photoelectron spectroscopy (XPS) (Fig. S12†). The crystallinity of the metathesized product was maintained and recognized to be isostructural with PCN-333-Fe(iii) as shown in the powder X-ray diffraction patterns (Fig. 3, bottom). The surface area and porosity of the metathesized product was also preserved based on the isotherms of N_2_ adsorption measurements (Fig. 3, top). These reaction conditions are optimized since higher or lower temperatures with longer or shorter reaction times will either yield partially metathesized material or cause structure decomposition and porosity loss.

The reductive labilization–metathesis process was facilitated in the PCN-333 system due to several structural characteristics. First, the microcrystalline and mesoporous nature of PCN-333 allows the metal ions to diffuse efficiently into the inner cavity of the framework. Besides, the trimeric clusters in PCN-333 are able to accommodate both di- and trivalent metal ions by varying the charges of the terminal ligands, the bridging oxygen atoms, and/or the number of counterions.^24^ Furthermore, the usage of the anhydrous reaction solvent decelerated the hydrolysis of the Cr(iii) species. The absence of acidic conditions contributes to the structural intactness of the intermediate MOF composed of Cr(ii)–O bonds.

In order to exclude the possibility that the metathesis of PCN-333-Fe(iii) with CrCl_2_ circumvented the reductive labilization mechanism, a redox inert isostructural framework PCN-333-Sc was synthesized as a template to metathesize with CrCl_2_. If the Cr(ii) species were hypothesized to metathesize with Fe(iii) ions in the framework directly without undergoing a redox reaction, complete metal metathesis should also be observed in the system of PCN-333-Sc and Cr(ii) since the Sc(iii)–O coordination bond is more labile than the Fe(iii)–O bond.^25^ The mixture of PCN-333-Sc and CrCl_2_ in dry DMF was heated at 85 °C for 30 minutes. The EDS results showed that only one fifth of the scandium in the framework was exchanged with chromium (Table S2†). This observation suggests that the metathesis between PCN-333-Fe(iii) and CrCl_2_ should proceed in a reductive labilization–metathesis manner since a more labile framework failed to generate a completely metathesized product without reductive labilization.

According to Marcus theory and some calculation results, the electron transfer between Fe(iii) and Cr(ii) in this case should follow an outer-sphere mechanism^27^ although the experimental evidence of this mechanism is still being pursued in our group. First of all, an inner-sphere mechanism requires the dissociation of axial ligands from Fe(iii) whereas an outer-sphere mechanism does not include coordination bond dissociation. Since the dissociation of Fe(iii)–ligand bonds is thermodynamically and kinetically unfavorable, an outer-sphere electron transfer mechanism is more reasonable. Moreover, based on calculations, the rate constant of the cross redox reaction between Fe(iii) and Cr(ii) in aqueous solution is nearly 3 × 10^7^ M^–1^ S^–1^, which is close to the rate constant of well-known outer-sphere redox couples, for example, [Fe(phen)_3_]^2/3+^ (1.5 × 10^7^ M^–1^ S^–1^),^27^ and much larger than well-known inner-sphere redox couples, for example, [Cr(H_2_O)_6_]^2+^ + [Co(NH_3_)_5_Cl]^2+^ (1.46 × 10^–2^ M^–1^ S^–1^). The metal ions are still in high-spin electronic configurations and their coordination field splittings in the framework or in DMF solutions are similar to those in aqueous solutions, which indicates that outer-sphere electron transfer is expected between these two metal ions.

As expected, the chemical stability of PCN-333-Cr(iii) is much more enhanced compared with that of PCN-333-Fe(iii). Suspended in water, HCl aqueous solution (pH = 0.0) and NaOH aqueous solution (pH = 11.0) at room temperature for 24 hours, PCN-333-Cr(iii) maintained structural integrity without an appreciable loss of crystallinity as confirmed from PXRD measurements (Fig. 3, bottom). In contrast, PCN-333-Fe(iii) was only stable in aqueous solutions at pH ranging from 3.0 to 9.0. To demonstrate the intactness of porosity, N_2_ isotherms were conducted before and after each treatment. The results indicated that the void volume accessibility, the characteristic mesoporous adsorption feature and the pore size distributions of PCN-333-Cr(iii) after each treatment were unequivocally preserved (Table S4, Fig. S3–S7†). Remarkably, the samples after each treatment even showed higher total adsorption amounts than the as-prepared PCN-333-Cr(iii). That is probably because some insoluble Cr(iii) compounds, generated during metal metathesis and trapped in the pores, were removed upon the above mentioned treatments.

The above results have clearly demonstrated that employing kinetically inert metal ions is an efficient strategy for constructing ultrastable MOFs with high porosity. Since the association–dissociation equilibrium of metal–ligand coordination bonds always exists, coordination bonds in a MOF also undergo an association–dissociation process. In aqueous solution, carboxylate ligand substitution around metal ions with other ligands from the solution, for example, water or hydroxyl groups, may take place, which could lead to the breakdown of the MOF structure. For two metal ions with the same valence, the ligand substitution rate of the kinetically inert species is far slower than that of the labile counterpart.^25^ As shown in Table 1, the ligand exchange rate of Cr(iii) in aqueous solution is much slower than that of the Fe(iii) species, which is believed to be the key factor that contributes to the superior chemical stability of Cr(iii)-MOFs compared with other MOFs based on trivalent species. Meanwhile, the slow Cr–ligand dissociation rate also decreases the hydrolysis rate of the carboxylate ligand which also contributes to the improvement of MOF stability.

**Table 1:** Ligand exchange rate of water molecules in trivalent aqua complexes^27^

	Inert complexes		Labile complexes		
M(OH_2_)	Cr(OH_2_)_6_^3+^	Ir(OH_2_)_6_^3+^	V(OH_2_)_6_^3+^	Fe(OH_2_)_6_^3+^	Ti(OH_2_)_6_^3+^
*k* (s^–1^)	2.4 × 10^–6^	1.1 × 10^–10^	8.7 × 10^1^	1.6 × 10^2^	1.8 × 10^5^

By taking advantage of the superior chemical stability of PCN-333-Cr(iii), alkylamine was incorporated in PCN-333-Cr(iii) with the aim of improving the CO_2_ adsorption capacity. Branched polyethylenimine (PEI, *M*_w_ = 800) was selected due to the high density of amine groups in each molecule. After PEI treatment, the solid maintained its crystallinity with a CO_2_ adsorption capacity of 8.4 wt% at 1 bar (Fig. S8†). In contrast, PCN-333-Fe(iii) completely lost its crystallinity and porosity after PEI treatment according to the PXRD pattern and N_2_ adsorption measurements (Fig. S9 and S10†).

## Conclusions

In conclusion, we reported a reductive labilization–metathesis route for the construction of an ultrastable mesoporous Cr(iii)-MOF from an iron based MOF template. The involvement of redox chemistry has switched a kinetically forbidden process into a feasible one. The whole process includes (1) reduction of Fe(iii) in the framework backbone to Fe(ii); (2) metal metathesis between Fe(ii) and Cr(ii); (3) oxidation of Cr(ii) in the framework to Cr(iii). The presence of an Fe(ii) intermediate was supported by the incomplete metathesis of PCN-333-Sc exchanged with CrCl_2_. After metathesis, PCN-333-Cr(iii) has demonstrated unprecedented chemical stability in aqueous solutions at pH 0.0 to 11.0 whereas PCN-333-Fe(iii) can only survive in solutions at pH 3.0 to 9.0. Significantly, PCN-333-Cr(iii) is robust enough to bear the harsh conditions of alkylamine solution, displaying a high CO_2_ adsorption capacity after PEI incorporation. Overall, the method we present here demonstrates a new platform to synthesize ultrastable MOFs with high porosity for practical applications.

## Experimental

### Materials and instruments

Iron(iii) chloride anhydrous (FeCl_3_), chromium(ii) chloride anhydrous (CrCl_2_), *N*,*N*-dimethylformamide (DMF), *N*,*N*-diethylformamide (DEF), trifluoroacetic acid (TFA) were purchased from Alfa Aesar. 4,4′,4′′-*s*-Triazine-2,4,6-triyltribenzoic acid (H_3_TATB) was synthesized following a reported procedure.^8^ All commercial chemicals were used without further purification.

Powder X-ray diffraction (PXRD) was carried out with a BRUKER D8-Focus Bragg-Brentano X-ray powder diffractometer equipped with a Cu sealed tube (*λ* = 1.54178 Å) at 40 kV and 40 mA. Thermogravimetric analyses (TGA) were conducted on a TGA-50 (SHIMADZU) thermogravimetric analyzer. Gas sorption measurements were conducted using a Micrometritics ASAP 2420 system at various temperatures. Inductively coupled plasma-mass spectrometry (laser ablation) was carried out using a Perkin-Elmer DRCII ICP-MS with both solution and laser ablation capabilities.

### Synthesis and activation of PCN-333-Cr(iii)

FeCl_3_ (60 mg), H_3_TATB (60 mg), DEF (10 mL) and TFA (0.5 mL) were mixed in a 20 mL vial. The solids were supersonically dissolved and the vial was heated at 150 °C for 12 hours. The resulting solid was centrifuged and washed several times with anhydrous DMF. 10 mL of anhydrous DMF was added into the vial and the mixture was degassed with nitrogen for 2 hours. 120 mg of CrCl_2_ was added into the vial in a glove box. Then the vial was heated at 85 °C for about 30 minutes until all of the solids turned green. Then the vial was centrifuged and transferred into the glove box to discard the mother liquor followed by rinsing three times with anhydrous DMF. The vial was taken out of the glove box and rinsed twice with DMF in the air. For sample activation, the sample was rinsed with acetone twice, dried in an 85 °C oven, and activated at 150 °C for 5 hours.

## Supplementary Material

Supplementary information
