# Supplementary material for: The preparation of an ultrastable mesoporous Cr(iii)-MOF via reductive labilization
Source: Chem Sci. 2015 Sep 2;6(12):7044–8. doi: 10.1039/c5sc02587g (PMC5947515; doi:10.1039/c5sc02587g)
Supplement: Supplementary file 1 [file SC-006-C5SC02587G-s001.pdf]

Electronic Supplementary Material (ESI) for Chemical Science.
This journal is © The Royal Society of Chemistry 2015

Supporting Information

The Preparation of An Ultrastable Mesoporous Cr(III)-MOF via Reductive Labilization

Xizhen Lian[†], Dawei Feng[†], Tian-Fu Liu[†], Qiang Zhang[†], Ying-Pin Chen[†,‡], Hong-Cai Zhou*[,†]

[†]Department of Chemistry, Texas A&M University, College Station, Texas 77842-3012, United States

[‡] Department of Material Science and Engineering, Texas A&M University, College Station, Texas 77843, United States

Contents:

S1. Stability test
S2. SEM-EDS analysis
S3. Metathesis of PCN-333-Fe(III) with $CrCl_3$
S4. Metathesis of PCN-333-Sc with $CrCl_2$
S5. ICP-MS result
S6. BET surface areas and DFT pore sizes for PCN-333-Fe(III), PCN-333-Cr(III) and PCN-333-Cr(III) after aqueous solution treatments
S7. Preparation of PEI-incorporated PCN-333-Cr(III) and PEI-incorporated PCN-333-Fe(III)
S8. CO_2 adsorptions of PEI-incorporated PCN-333-Cr(III) and PEI-incorporated PCN-333-Fe(III)
S9. PXRD patterns of PEI-incorporated PCN-333-Cr(III) and PEI-incorporated PCN-333-Fe(III)
S10. N_2 isotherms of PEI-incorporated PCN-333-Cr(III) and PEI-incorporated PCN-333-Fe(III)
S11. Thermogravimetric analysis of PCN-333-Cr(III)
S12. XPS result of PCN-333-Cr(III)
S13. Rate constant calculations according to Marcus Theory

S1. Stability test

60 mg solid was suspended in 10 mL aqueous solution at different pH values for 24 hours under room temperature. The solid was collected by centrifuge and was rinsed by acetone three times, dried at 85 ^{o}C, and activated at 150 ^{o}C for 5 hours.

S2. SEM-EDS analysis

Metathesis between PCN-333-Fe(III) and $CrCl_2$

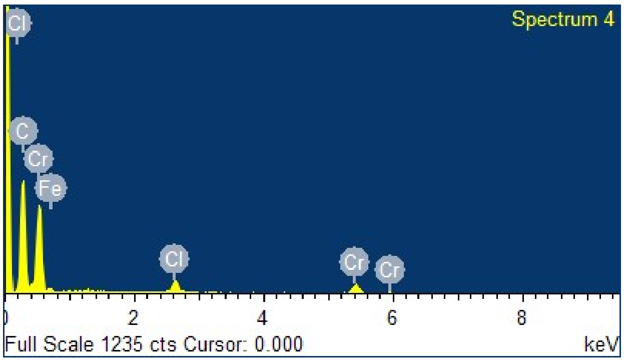

Figure S1 EDS result of metathesis between PCN-333-Fe(III) and $CrCl_2$

Table S1. EDS result of metathesis between PCN-333-Fe(III) and $CrCl_2$

Element	Weight%	Atomic%
C K	42.49	75.75
Cl K	3.60	2.17
Cr L	49.42	20.35
Fe L	4.49	1.72
Totals	100.00	

Table S2. Metathesis between PCN-333-Sc and $CrCl_2$

Element	Weight%	Atomic%
C K	52.40	67.93
O K	24.68	24.02
Cl K	4.41	1.94
Sc K	12.24	4.24
Cr L	6.27	1.88
Totals	100.00	

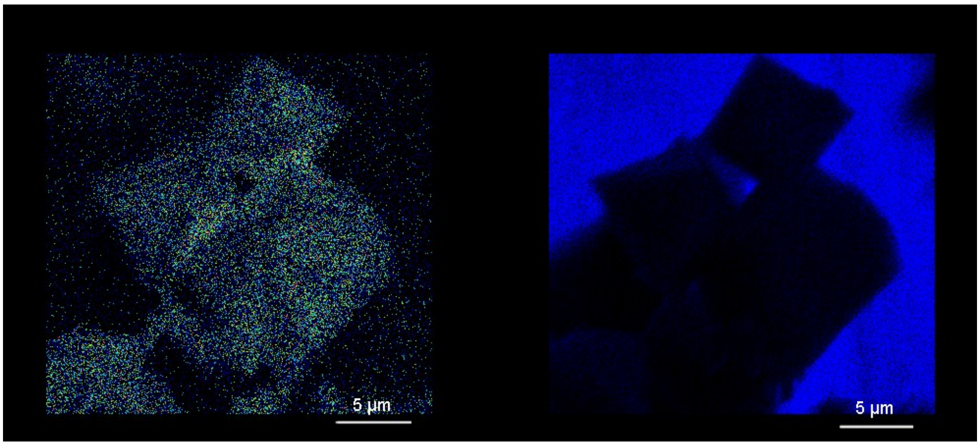

Figure S2 SEM-EDS mapping picture of Cl (left), and Cu (right), of PCN-333-Cr(III). The substrate was made of Cu.

S3. Metathesis of PCN-333-Fe(III) with $CrCl_3$

120 mg $CrCl_3$ was dissolved in 10 mL DMF under rigorous stirring and heating. Freshly prepared PCN-333-Fe(III) was added into the above solution. The mixture was warmed at 85 ^oC for 24 hours. The solid was collected by centrifuge and washed with DMF.

S4. Metathesis of PCN-333-Sc with $CrCl_2$

PCN-333-Sc was synthesized following the procedure in the literature.[1] 10 mg of freshly prepared PCN-333-Sc was suspended in 2 mL DMF in a 4 mL pyrex vial. 30 mg $CrCl_2$ was added into the vial in a glove box. The mixture was warmed at 85^oC for 20 minutes and the solid was collected by centrifuge and washed with DMF.

S5. ICP-MS results

Each sample was measured three times. The chart only showed the average value of the results for each sample.

Table S3. ICP-MS results of each metathesis

Sample	Metal 1	Concentration /ppb	Metal 2	Concentration /ppb	Metal 1: Metal 2 /molar ratio
1	Cr	869.11	Fe	72.92	12: 1
2	Cr	454.85	Fe	132.38	3.44: 1

Sample 1: PCN-333-Fe(III) exchanged by $CrCl_2$. Sample 2: PCN-333-Fe(III) exchanged by $CrCl_3$.

S6. BET surface areas and DFT pore sizes for PCN-333-Fe(III), PCN-333-Cr(III) and PCN-333-Cr(III) after aqueous solution treatments

Table S4. BET surface areas and DFT pore sizes summary

	BET surface area /m^2 g^{-1}	Total Volume in Pores /cm^3 g^{-1}	Total Area in Pores /m^2 g^{-1}
PCN-333-Fe(III)	2427	2.72	1603
PCN-333-Cr(III)	2548	2.30	1611
PCN-333-Cr(III) treated with water	2742	2.69	1759
PCN-333-Cr(III) treated with pH=0 solution	2678	2.66	1717
PCN-333-Cr(III) treated with pH=11 solution	2610	2.54	1656

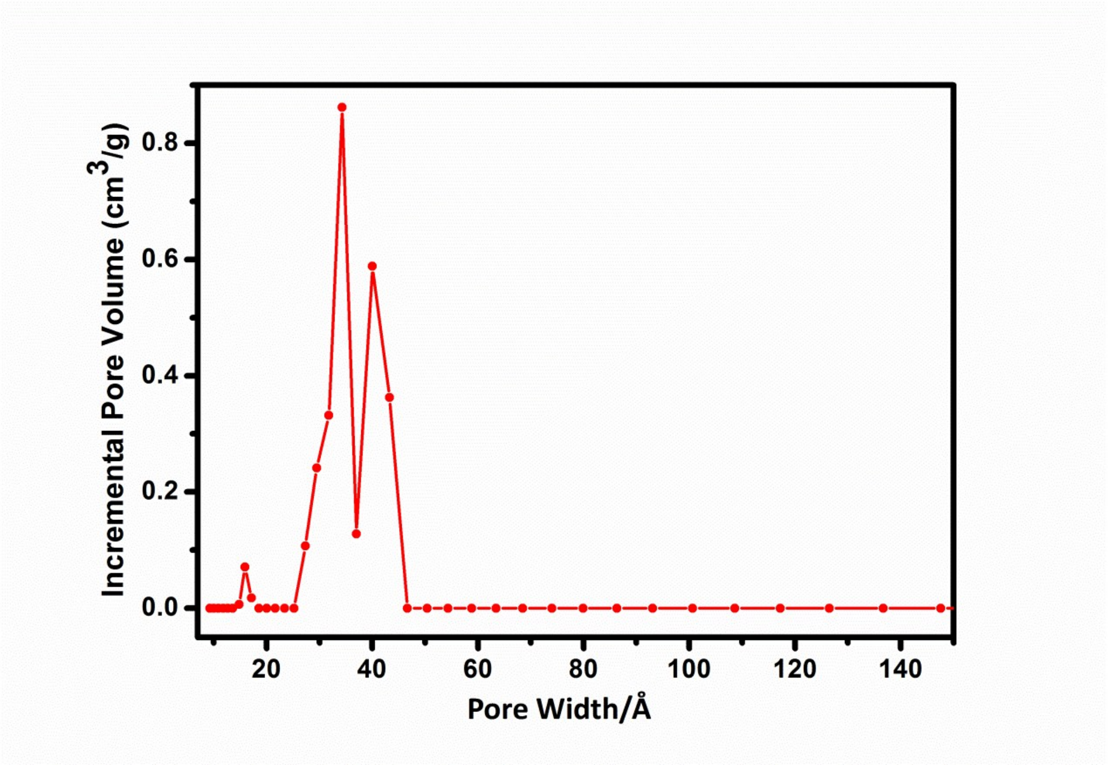

Figure S3 Pore size distribution of PCN-333-Fe(III)

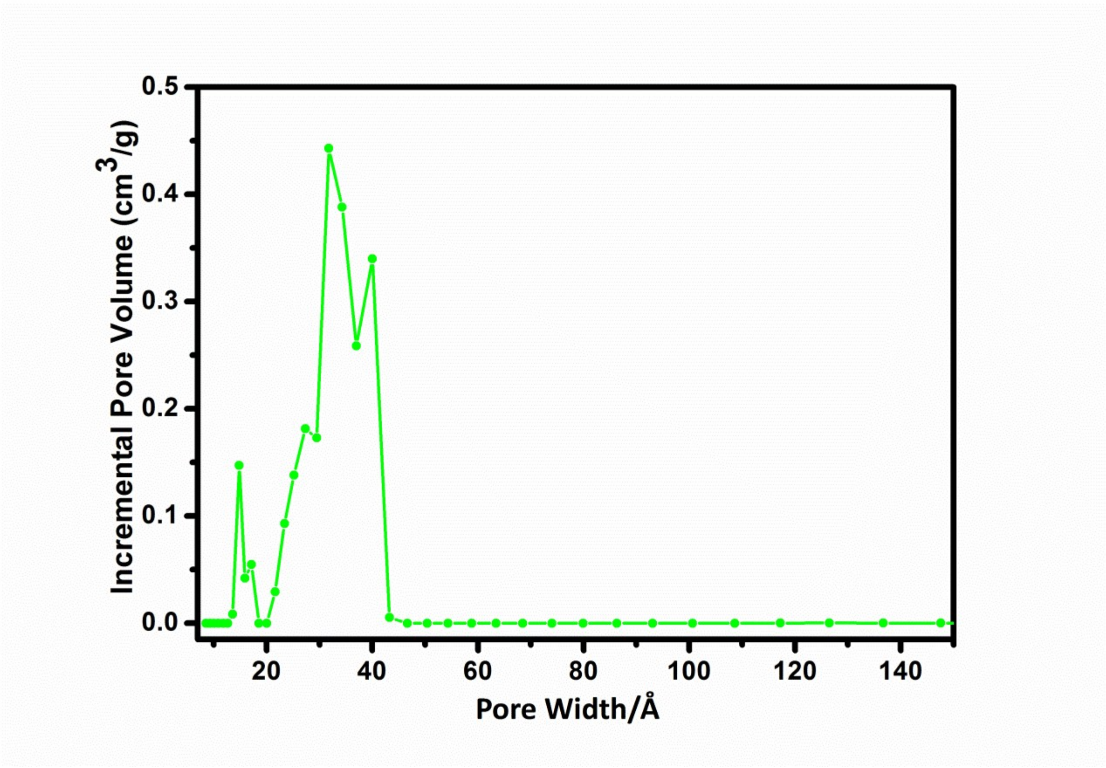

Figure S4 Pore size distribution of PCN-333-Cr(III)

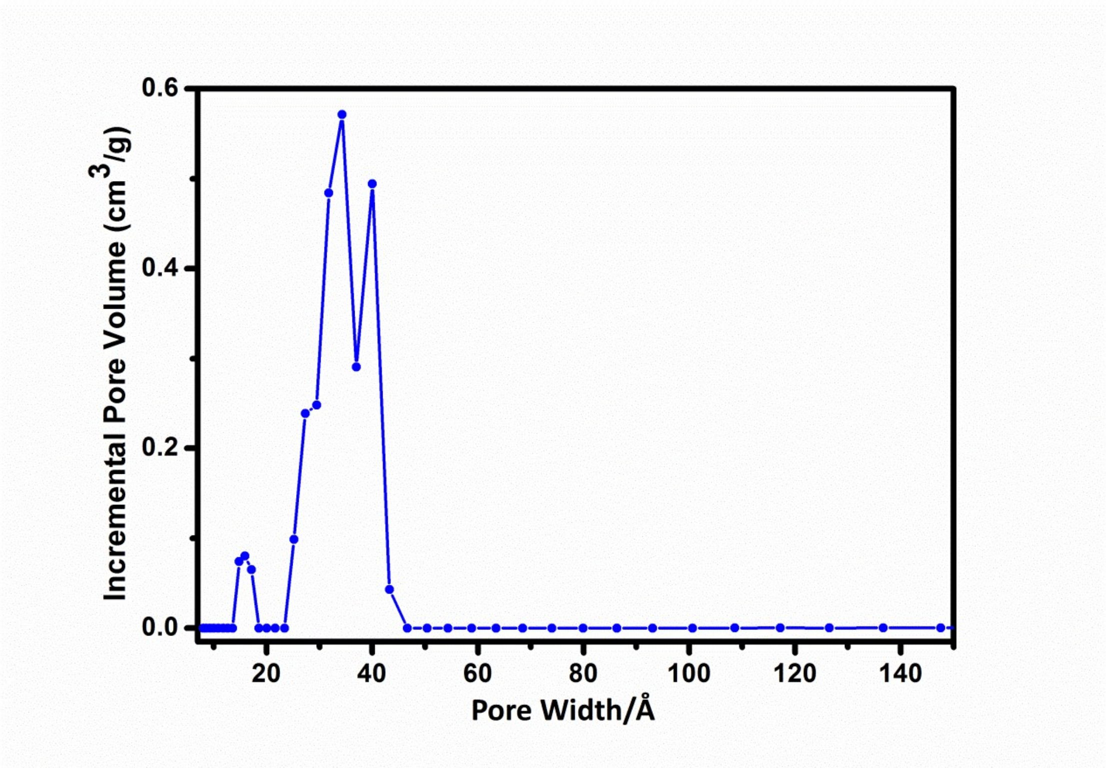

Figure S5 Pore size distribution of PCN-333-Cr after water treatment

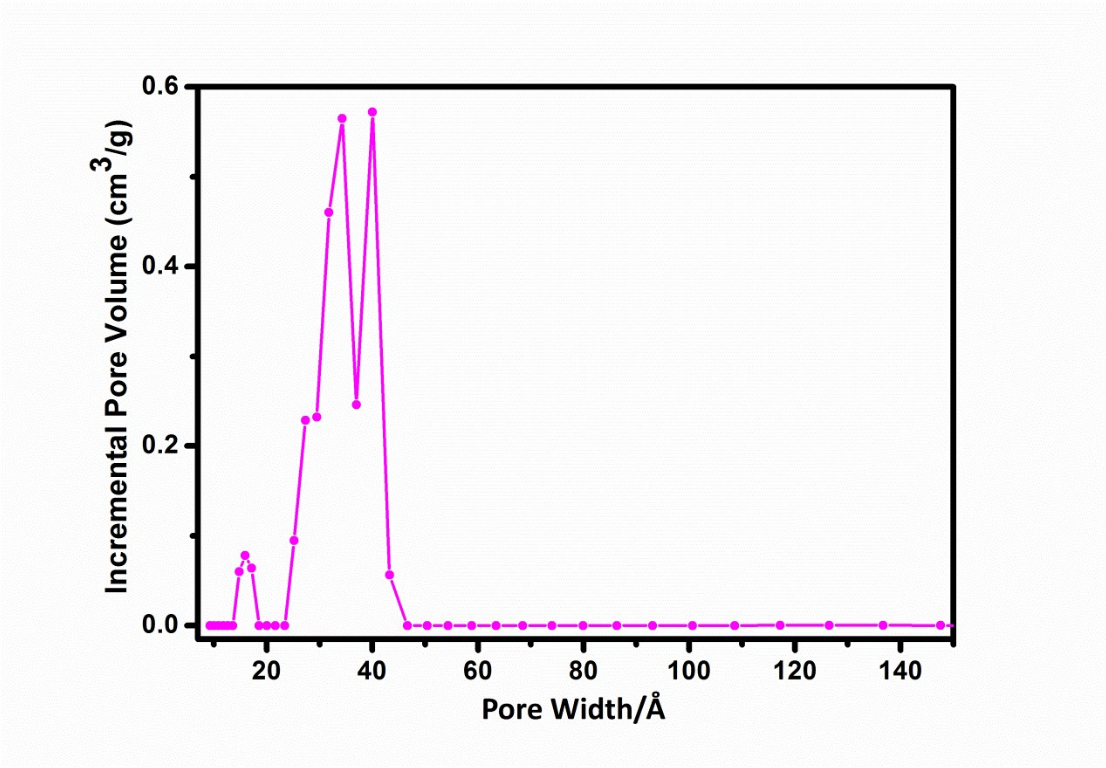

Figure S6 Pore size distribution of PCN-333-Cr(III) treated with pH=0 solution

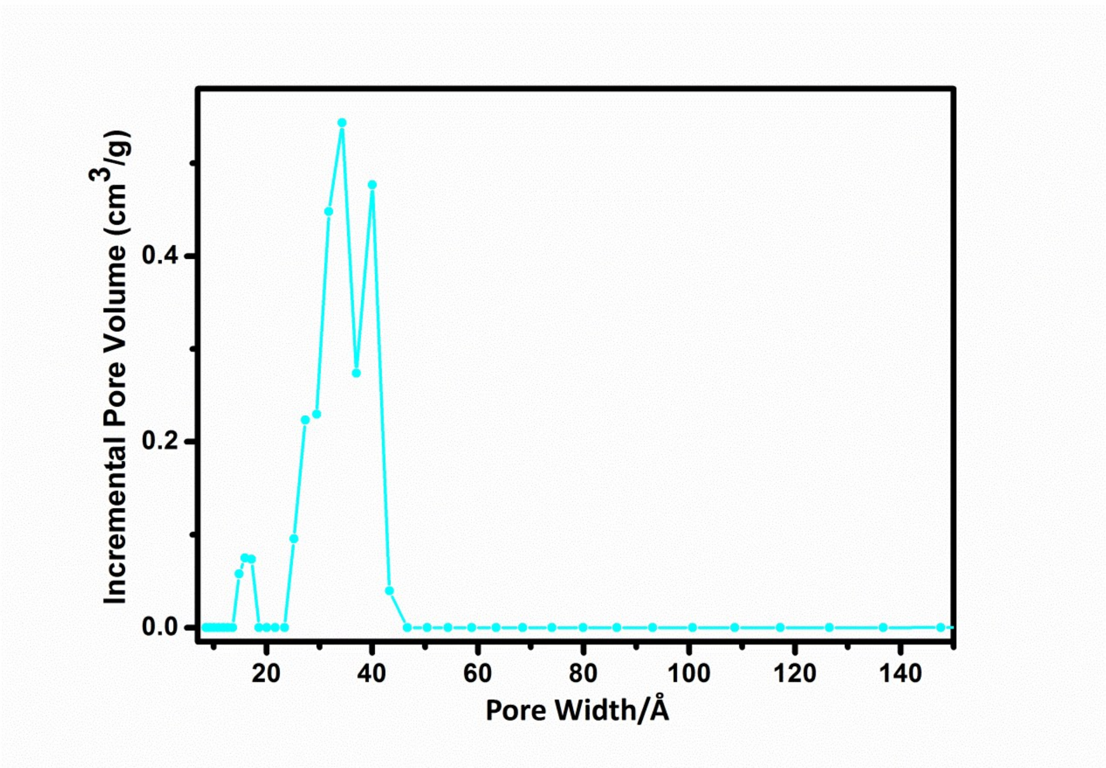

Figure S7 Pore size distribution of PCN-333-Cr(III) treated with pH=11 solution

S7. Preparation of PEI-incorporated PCN-333-Cr(III) and PEI-incorporated PCN-333-Fe(III)

60 mg activated PCN-333-Cr(III) was suspended in anhydrous dichloromethane (5mL) and 300 mg PEI was slowly added in the slurry. The mixture was well mixed by gentle shaking for 20 minutes. The solid was separated by centrifuge and the excess PEI was washed by dichloromethane. The sample was first dried under vacuum and activated at 80 °C for 1 hour. PEI-incorporated PCN-333-Fe(III) was obtained in the same manner as PEI-incorporated PCN-333-Cr(III).

S8. CO_2 adsorptions of PEI-incorporated PCN-333-Cr (III) and PEI-incorporated PCN-333-Fe (III)

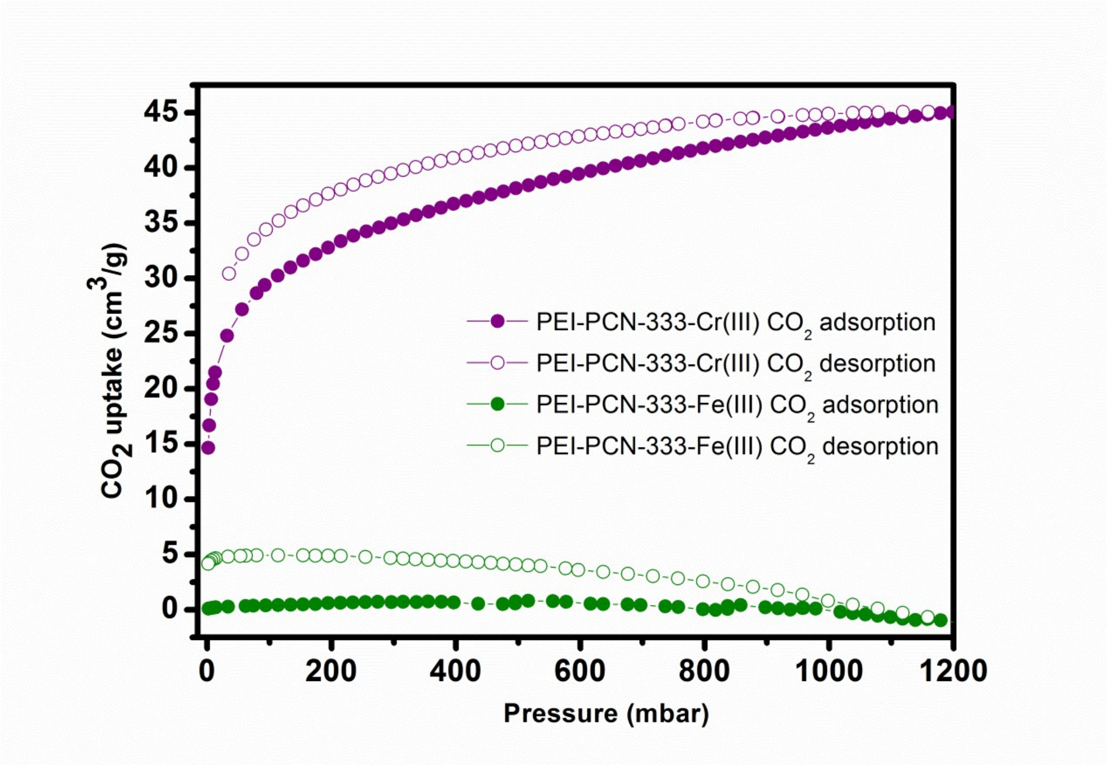

Figure S8 CO_2 adsorptions of PEI-incorporated PCN-333-Cr(III) and PEI-incorporated PCN-333-Fe(III)

S9. PXRD patterns of PEI-incorporated PCN-333-Cr(III) and PEI-incorporated PCN-333-Fe(III)

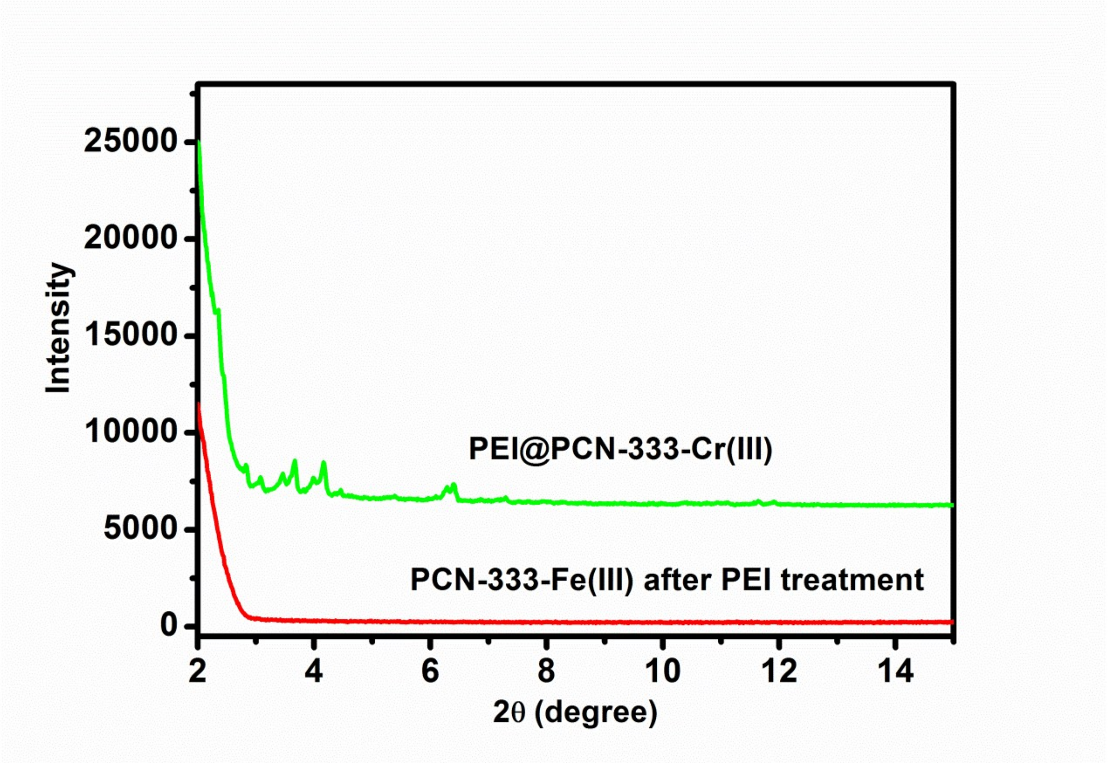

Figure S9 PXRD patterns of PEI-incorporated PCN-333-Cr(III) and PEI-incorporated PCN-333-Fe(III)

S10. N_2 isotherms of PEI-incorporated PCN-333-Cr(III) and PEI-incorporated PCN-333-Fe(III)

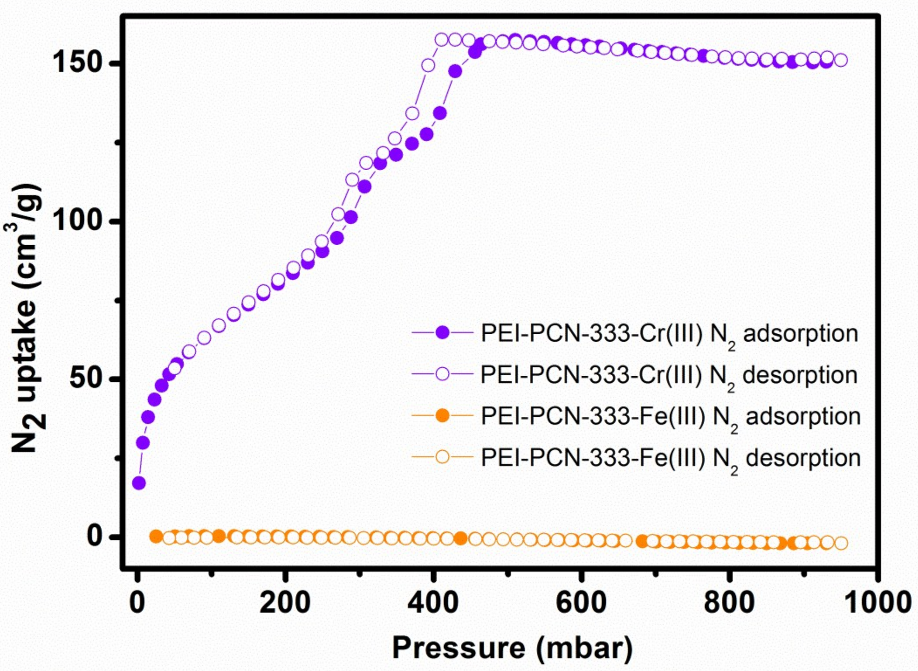

Figure S10 N_2 isotherms of PEI-incorporated PCN-333-Cr(III) and PEI-incorporated PCN-333-Fe(III)

S11. Thermogravimetric analysis of PCN-333-Cr(III)

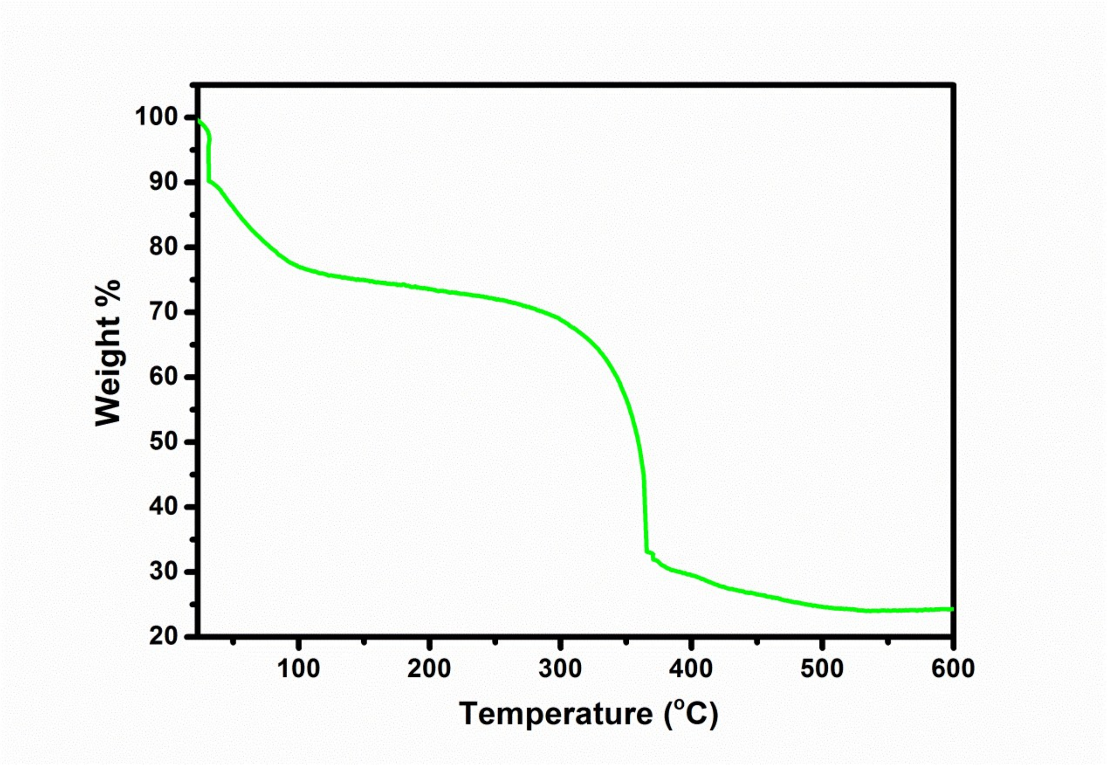

Figure S11 Thermogravimetric analysis of PCN-333-Cr(III)

S12. XPS result of PCN-333-Cr(III)

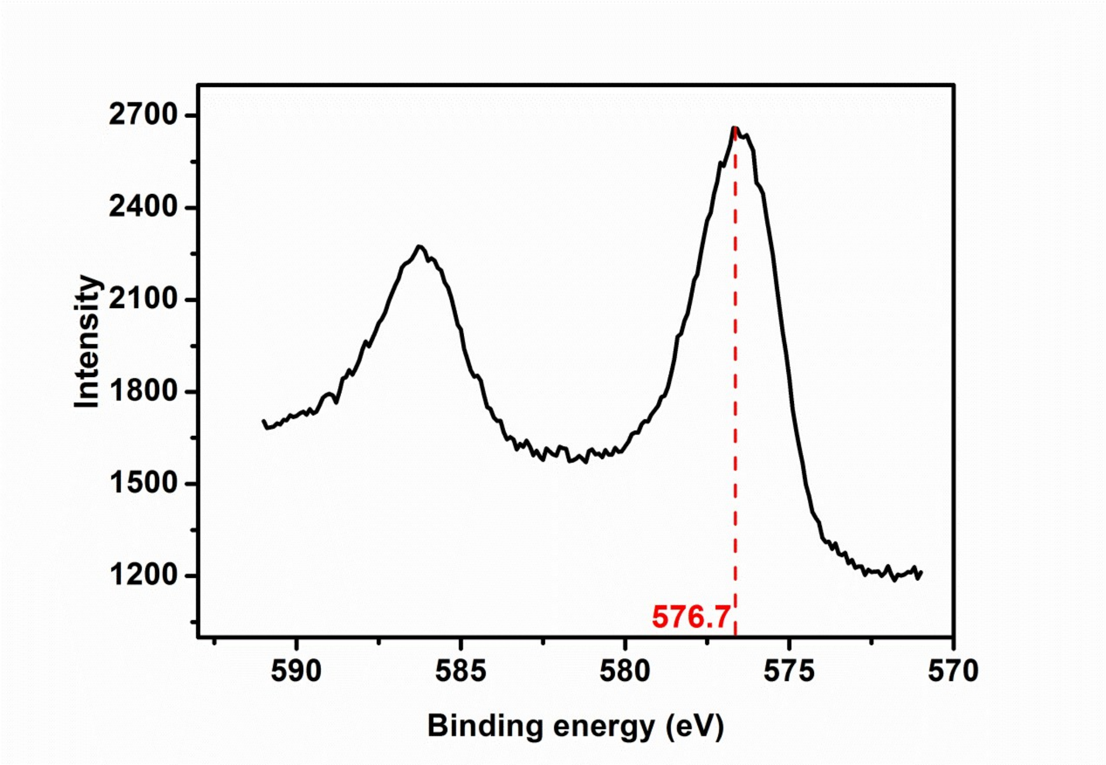

Figure S12 XPS result of PCN-333-Cr(III). The peak at 576.7 eV indicates the presence of Cr(III).

S13. Rate constant calculations according to Marcus Theory

$$k_{AB} = (k_{AA}k_{BB}K_{AB}f_{AB})^{1/2} \quad f_{AB} = \frac{(logK_{AB})^2}{4log\frac{k_{AA}k_{BB}}{Z^2}} \approx 1$$

k_{AB} = rate of cross reaction; k_{AA}, k_{BB} = self exchange rates; K_{AB} = equilibrium constant of reaction; Z = collision frequency for hypothetical uncharged complex (10^{11}-$10^{13}\ M^{-1}s^{-1}$).

$$k_{AB} \approx (k_{AA}k_{BB}K_{AB})^{1/2}$$

$$Fe^{3+} + Cr^{2+} \rightarrow Fe^{2+} + Cr^{3+}$$

$$Fe^{3+} + e^{-} \rightarrow Fe^{2+} \qquad E^0 = 0.77\ V$$

$$Cr^{3+} + e^{-} \rightarrow Cr^{2+} \qquad E^0 = -0.40\ V$$

$$Fe^{3+} + Fe^{2+} \rightarrow Fe^{2+} + Fe^{3+} \qquad k_{AA} = 4.5\ M^{-1}s^{-1}$$

$$Cr^{3+} + Cr^{2+} \rightarrow Cr^{2+} + Cr^{3+} \qquad k_{BB} = 1 \times 10^{-5}\ M^{-1}s^{-1}$$

$$\Delta G^0 = -RTlnK$$

$$\Delta G^0 = -nF\Delta E^0$$

$$-RTlnK = -nF\Delta E^0 \quad K = e^{\frac{nF\Delta E^0}{RT}}$$

$$K_{AB} = e^{38.94(0.77 + 0.40)} = 1.92 \times 10^{19}$$

$$k_{AB} = (1.92 \times 10^{19} \times 4.5 \times 1 \times 10^{-5})^{1/2} = 2.94 \times 10^7\ M^{-1}s^{-1}$$

The calculation of $[Cr(H_2O)_6]^{2+}$+$[Co(NH_3)_5Cl]^{2+}$ couple is in the same manner.

Reference

(1) Jordan, R. B. Reaction Mechanisms of Inorganic and Organometallic Systems; OUP Oxford, 2007.
